# Supplementary material for: A time-varying analysis between economic uncertainty and tourism development in Singapore
Source: PLoS One. 2024 May 24;19(5):e0302980. doi: 10.1371/journal.pone.0302980 (PMC11125502; doi:10.1371/journal.pone.0302980)
Supplement: Attachment — Submitted filename: Response to Editor-in-Chief & Reviewers.docx [file pone.0302980.s001.docx]

**Overall Modifications**

The authors thank Prof. Ricky Chee Jiun Chia - the Academic Editor of Plos One journal, and the two esteemed reviewers for their valuable comments. We regret some weaknesses of the original manuscript. In this revision, The authors have tried to address every comment and suggestion. All modified parts are indicated in **green** in the new version of the manuscript. The authors sincerely hope that the revised manuscript meets the expectations of the Editors and Reviewers.

**Response to Reviewer 1**

Q1. The abstract is too plain and needs to inform the reader more on the obtained results and policy implications/importance of the study.

A1. The experience and sharing of reviewers is highly appreciated. We have tried to improve the abstract section. We hope that it makes the contribution clear. Please see the Abstract in the revised manuscript or the below text:

“Tourism development (TO) is seen as a viable solution to address economic policy uncertainty (EPU) risks. However, previous studies have largely ignored the relationship between short, medium, and long term by decomposing TO and EPU index at different time-frequency scales, especially in Singapore. In this study, the Wavelet tools analysis and a rolling window algorithm are employed to re-visit the causal relationship between EPU, industrial production index (IPI), government revenue (GR), and tourism development (TO) in Singapore from January 2003 to February 2022. The findings revealed the heterogeneous effects of EPU on TO at different time horizons in terms of importance and magnitude over time. A rise in EPU results in a decline in TO at the low frequencies, indicating that EPU has a detrimental effect on TO over the short term. Conversely, in the long term, an increase in TO results in a decrease in EPU. Furthermore, the outcome also indicated that there is a uni-directional causality running from TO to EPU, GR and IPI. Expressly, we confirm that the negative co-movement is more pronounced in the aftermath of the COVID-19 crisis, particularly for EPU, and GR at low-medium frequencies throughout the research period. The findings provide tourism policymakers with insight to develop strategic plans for tourism development that consider the effects of economic policy uncertainty. By understanding how uncertainty impacts tourism, governments can tailor development strategies to mitigate risks and capitalize on opportunities”

Q2. In the literature review, whilst the review of empirical studies is sufficient, the theoretical foundations of the study is missing.

A2. Thanks for this comment. To our best knowledge, the theoretical foundations reflect that the direct association between tourism development and economic policy uncertainty is missing. Instead of showing theories, we summarized previous studies to better highlight a gap in the existing literature and the specific contribution of this research. Please see pages 5-7 in the revised manuscript for more details.

Q3. The methods need to be described in more detailed. For instance, in the wavelet coherence analysis, what is the ‘mother wavelet’ that is being used and why was this mother wavelet chosen? Also, the time-varying causality tests need to be described in greater detail. What are the null hypothesis of the causality tests and what criteria are used for rejecting or accepting the tested hypotheses?

A3. We fully agree with this comment. The methods were described in more detail. We followed the suggestion of Torrence and Webster (1999) to decompose examined variables. More precisely, the 2 to 8-month scale presents the short-run horizon. Similarly, 8 to 32 months refers to medium-term horizon, and the period above 32 months demonstrates the long-run horizon.

Moreover, we added the null hypothesis for the time-varying causality test, and criteria were used to reject the tested hypothesis. Please see pages 11-12 for more details.

Q4. In the empirical results, the authors need to show the results from the wavelet power spectrum plots to give an indication the time-frequency evolution of the individual time series which would be of interest to the readers. This can be provided together with some basic time series statistics for the variables. Also, unit root test results are important as the time-varying causality tests require the series to be stationary. Collectively, the authors should include a new section addressing these issues. The wavelet coherence plots do not report the actual dates on the horizontal axis. The authors need to do so as it is difficult to interpret the wavelet plots without knowing when ‘certain events’ took place.

A4. Thanks for this comment. We updated Figure 1, Table 1, and Table 2 to summary our results. Furthermore, the unit root test was added, and the empirical result has been modified to make our findings clear. Please see pages 12-17 for more details.

Q5. The comparison between the wavelet coherence plots and time-varying causality tests has not been done properly. What are the common findings and what findings differ? Are there an explanations for the differences in the findings.

A5. Thanks for this comment. There are some differences between two econometric approaches, so it is difficult to comparison findings. However, we try to indicate several “certain events” findings of two techniques. It helps to make our findisngs clear. Overall, the TO variable demonstrates a strong correlation with EPU, a moderate correlation with GR, and a weaker correlation with the IPI variable. Please see pages 16-17 for more details.

Q6. In the further discussion of the results, much emphasis was place on the economic uncertainty-tourism relationship and not much was said about the government- expenditure-tourism or industrial production-tourism relationships. The authors need to discuss these three speres collectively. Same applies to the conclusion of the paper.

A6. We fully agree with this comment. The discussion section has been modified to compare our findings to those of relevant prior studies. It makes our contribution clear. Please see pages 18-20 for more details, or below text:

“The results of this study shed light on the nexus between economic policy uncertainty, government revenue, industrial production index, and tourism development in Singapore from January 2003 to February 2022. Despite the abundance of previous studies, the relationship between EPU and tourism development still has much attention from economists and policymakers worldwide. By applying Wavelet tools, the findings of this study confirmed a robust negative relationship between EPU and TO in Singapore from January 2003 to February 2022 at different time-frequency scales. More precisely, economic uncertainty negatively affects tourism development at low frequencies from February 2013 to August 2018. In contrast, in the long term, the Wavelet coherence plots revealed that an increased tourism development leads to decreased EPU. The findings align with the conclusions of Wu and Wu [46] for BRIC countries and Singh, Daset al. [28] for the United States economy. Singapore's economy is highly interconnected with the global economy. Events such as the Global Financial Crisis (2008-2009), the European Debt Crisis (2010-2012), and the COVID-19 pandemic (2020 onwards) likely contributed to periods of heightened economic policy uncertainty in Singapore. Uncertainties surrounding trade tensions between major economies, geopolitical events, and shifts in global economic trends could also influence economic policy uncertainty in Singapore. When facing economic uncertainty, the priority choice of individuals and enterprises is decreasing investments and reducing outbound tourism expenditures, which negatively affect tourism investments, hindering the growth of the tourism sector. An intriguing finding the authors realize is that increasing tourism development reduces EPU.

Some reasons are suggested to explain the findings: (i) In the short term, tourism is susceptible to various forms of uncertainty, such as conflicts, terrorism, natural catastrophes, epidemics, contagions, and financial crises, which can negatively impact tourism demand [47, 48]. (ii) In the long term, developing the tourism industry will stimulate the diversification of national revenue sources. When the economy depends on many sectors, fluctuations in a specific sector will not strongly impact the entire economy [33]. Additionally, developing the tourism industry also strengthens local economies [49], boosts exporting [50], enhances national image [37], and creates community stability [51]. Hence, many economists and policymakers believe that boosting tourism development is crucial to combat economic uncertainty.

Another important finding of this study is that tourism development can lead to a reduction in the development of other sectors in Singapore in the short- and medium-term. In contrast, TO tends to enhance IPI in the long term. Significant investments in tourism infrastructure, such as hotels, entertainment facilities, and tourist attractions, may divert resources away from other sectors [52]. This allocation of resources could limit funding available for the development of other industries. Besides, a heavy reliance on tourism revenue may lead to a disproportionate focus on catering to tourists' needs and preferences at the expense of other sectors. This focus can skew economic priorities and neglect the development of diverse industries. Fortunately, this conclusion is only valid for the period 2003 – 2015. According to the World Travel & Tourism Council [53], the Singaporean government prioritized investments and resources in the tourism sector at the expense of other sectors from 1978 to 1992. This results in insufficient funding for areas such as education, healthcare, or infrastructure. Realizing this limitation, after 2015 the Singapore government applied many measures to diversify the economy, focusing on digital transformation, high technology, and financial services. These policies help Singapore better respond to risks and promote the supporting role of tourism development for other sectors. This conclusion is supported by Li, Duanet al. [54], who confirmed that tourism development efficiency of 58 China’s cities is increasing year by year.

In addition, the authors discover that initially, a rise in tourism activity corresponds to a rise in government revenue. However, the correlation between tourism and government revenue has become ambiguous after January 2013. This suggests that the Singaporean government has effectively broadened its revenue streams and diminished its dependence on the tourism sector. In other words, while tourism continues to contribute to government revenue, other sectors or sources of revenue have become increasingly important. This diversification strategy is beneficial because it reduces Singapore's vulnerability to fluctuations or downturns in the tourism industry. It shows that Singapore's economy has matured and diversified, allowing the government to maintain stable revenue streams even when tourism may not be performing as strongly. By investing in and promoting other sectors, such as finance, technology, and manufacturing, Singapore could have expanded its revenue sources, leading to a weaker correlation between tourism and government revenue. This outcome contrasts sharply with viewpoint of Gnangnon [55]. The empirical findings demonstrate that international tourism receipts have a notable and beneficial influence on non-resource tax revenue from 1995 to . Moreover, this influence strengthens as countries progress in development, suggesting that advanced economies benefit more from increased international tourism receipts in terms of non-resource tax revenue compared to less developed economies.”.

Finally, some new articles were added. Please see the green highlights in the reference section for more details.

**Response to Reviewer 2**

Q1. In the introduction it is not clear if authors are considering impact of tourism on policy uncertainty or vice versa. In general, from the introduction, the same lack of clarity involves all the causality links they are investigating. It should be clarified from the start that they are exploring both directions of causality for all three of the investigated variables as this appears to be their main contribution.

A1. Yes, we fully agree with this comment. In the revised manuscript, we showed that the motivations for implementing. Moreover, we also modified the Abstract to make our contribution clear. Please see the pages 1-3 for more details, or below text:

“Tourism development (TO) is seen as a viable solution to address economic policy uncertainty (EPU) risks. However, previous studies have largely ignored the relationship between short, medium, and long term by decomposing TO and EPU index at different time-frequency scales, especially in Singapore. In this study, the Wavelet tools analysis and a rolling window algorithm are employed to re-visit the causal relationship between EPU, industrial production index (IPI), government revenue (GR), and tourism development (TO) in Singapore from January 2003 to February 2022. The findings revealed the heterogeneous effects of EPU on TO at different time horizons in terms of importance and magnitude over time. A rise in EPU results in a decline in TO at the low frequencies, indicating that EPU has a detrimental effect on TO over the short term. Conversely, in the long term, an increase in TO results in a decrease in EPU. Furthermore, the outcome also indicated that there is a uni-directional causality running from TO to EPU, GR and IPI. Expressly, we confirm that the negative co-movement is more pronounced in the aftermath of the COVID-19 crisis, particularly for EPU, and GR at low-medium frequencies throughout the research period. The findings provide tourism policymakers with insight to develop strategic plans for tourism development that consider the effects of economic policy uncertainty. By understanding how uncertainty impacts tourism, governments can tailor development strategies to mitigate risks and capitalize on opportunities.”

Q2. In the introduction the order of issues as presented in points i), ii) and iii) in the introduction is not consistent with the more detailed description that follows.

A2. Yes, we followed this comment to revise original manuscript. Please see the Literature review section for more details.

Q3. Since the dataset also includes the Covid-19 pandemic period, the authors should better comment on how this may affect their results. For example, could the fact there are no long-term relationships between TO and EPU be a consequence of the inclusion of pandemic data? Perhaps running the analysis excluding 2020-22 could be a robustness check.

A3. Yes, we agree with this comment. However, both cross-wavelet coherence and time-varying causality techniques require long data. We divided data into two periods and focused on the period of the COVID-19 pandemic. However, the length of data for the time of the COVID-19 pandemic does not satisfy the condition of the two techniques.

Q4. Sometimes the authors use economic stability and EPU as synonyms, but they are not.

A4. Yes, this error has been edited in the revised manuscript.

Finally, some typing, grammatical, and structural errors were found and edited. The authors sincerely hope that the revised manuscript meets the expectations of the Academic Editor and Reviewer.
